# The COX- inhibitor indomethacin reduces Th1 effector and T regulatory cells in vitro in Mycobacterium tuberculosis infection

**DOI:** 10.1186/s12879-016-1938-8

**Published:** 2016-10-24

**Authors:** Kristian Tonby, Ida Wergeland, Nora V. Lieske, Dag Kvale, Kjetil Tasken, Anne M. Dyrhol-Riise

**Affiliations:** 1 Institute of Clinical Medicine, University of Oslo, Oslo, Norway; 2 Department of Infectious Diseases, Oslo University Hospital, Oslo, Norway; 3 Department of Clinical Science, University of Bergen, Bergen, Norway; 4 Centre for Molecular Medicine Norway, Nordic EMBL Partnership, University of Oslo, Oslo, Norway; 5 K.G. Jebsen Inflammation Research Centre, University of Oslo, Oslo, Norway; 6 Biotechnology Centre, University of Oslo, Oslo, Norway

**Keywords:** Tuberculosis, COX-inhibitors, Tregs, Regulatory T cells, Host-directed therapy, Monocytes, Cytokines

## Abstract

**Background:**

Tuberculosis (TB) causes a major burden on global health with long and cumbersome TB treatment regimens. Host-directed immune modulating therapies have been suggested as adjunctive treatment to TB antibiotics. Upregulated cyclooxygenase-2 (COX-2)-prostaglandin E2 (PGE2) signaling pathway may cause a dysfunctional immune response that favors survival and replication of *Mycobacterium tuberculosis (Mtb)*.

**Methods:**

Blood samples were obtained from patients with latent TB (*n* = 9) and active TB (*n* = 33) before initiation of anti-TB chemotherapy. COX-2 expression in monocytes and ESAT-6 and Ag85 specific T cell cytokine responses (TNF-α, IFN-γ, IL-2), proliferation (carboxyfluorescein succinimidyl ester staining) and regulation (FOXP3+ T regulatory cells) were analysed by flow cytometry and the in vitro effects of the COX-1/2 inhibitor indomethacin were measured.

**Results:**

We demonstrate that indomethacin significantly down-regulates the fraction of *Mtb* specific FOXP3+ T regulatory cells (ESAT-6; *p* = 0.004 and Ag85; *p* < 0.001) with a concomitant reduction of *Mtb* specific cytokine responses and T cell proliferation in active TB. Although active TB tend to have higher levels, there are no significant differences in COX-2 expression between unstimulated monocytes from patients with active TB compared to latent infection. Monocytes in both TB groups respond with a significant upregulation of COX-2 after in vitro stimulation.

**Conclusions:**

Taken together, our in vitro data indicate a modulation of the Th1 effector and T regulatory cells in *Mtb* infection in response to the COX-1/2 inhibitor indomethacin. The potential role as adjunctive host-directed therapy in TB disease should be further evaluated in both animal studies and in human clinical trials.

## Background

Infection with *Mycobacterium tuberculosis (Mtb)* causes a major burden on global health with 9 million people suffering from tuberculosis (TB) disease and with 1.5 million deaths every year [1]. The current TB treatment strategies consist of long lasting multiple drug regimens with risk of serious side-effects and development of multi-drug resistant TB (MDR-TB). Host-directed therapies (HDTs) in conjunction with standard anti-TB drug regimens may reduce the duration of therapy, achieve better treatment outcomes, lower the risk of developing further drug resistance and decrease the chances of relapse or reinfection [2, 3].

In chronic infections such as TB, immune-mediated tissue injury may become more detrimental than the pathogen itself and the immune system have evolved mechanisms to balance pro and anti-inflammatory signals [4]. FOXP3+ T regulatory cells (Tregs) are involved in the regulation of inflammatory processes and exert immunosuppressive functions by cell contact-dependent suppression of CD4+ T cells and by secretion of inhibitory cytokines and soluble factors [5, 6]. Tregs may dampen protective immunity facilitating pathogen multiplication and dissemination [7] and may also limit vaccine immunogenicity [8]. Thus, targeting of Tregs may have potential as host directed adjunctive therapies [9].

Prostaglandin E2 (PGE2) is generated by the constitutive cyclooxygenase 1 (COX-1) and the inducible cyclooxygenase 2 (COX-2) enzymes and is regarded as a key mediator of immunopathology with immune regulatory effects in chronic infections [10, 11]. Macrophages and monocytes up-regulate COX-2 enzymes in response to inflammatory signals and are thereby major producers of PGE2 and other eicosanoids [12]. Monocytes as well as adaptive Tregs seem to inhibit effector T cell functions and suppress T cell immune responses by a COX-2-PGE2-dependent mechanism [13–15]. It has been shown that highly expressed COX-2 in malignant tissue is associated with poor prognosis and outcome in cancer disease [16, 17]. However, no data exists on COX-2 expression of immune cells in human TB disease.

Standard TB antibiotics are directed against the pathogen, but various host directed immune therapies, including reduction of PGE2 production by COX- inhibitors (COX-i) has potential to become part of a treatment strategy for resistant or clinically complicated TB cases or as part of a TB vaccination scheme [18–20]. Accordingly, studies of TB animal models have shown that targeting PGE2 with COX-i have significant impact on the immune responses and outcome of disease [21–25]. Based on these data, it is suggested that a human intervention study with anti-inflammatory drugs given in combination with anti-TB chemotherapy should be performed [24]. Clinical trials in HIV infected patients have also shown that COX-i improve T cell mediated immune responses [26–28].

Indomethacin is a widely used non-steroidal anti-inflammatory drug (NSAID) of the methylated indole class with analgesic and antipyretic properties exerting its pharmacological effects by inhibiting the synthesis of prostaglandins via the arachidonic acid pathway [29]. Indomethacin inhibits both COX-1 and COX-2 with greater selectivity for COX-1 [30] and due to water soluble characteristics the compound is practical to use in in vitro studies. Indomethacin has previously been shown to increase the bactericidal activity of *Mtb* infected macrophages [31] and to improve T cell proliferative responses in HIV-infected patients [28], but to our knowledge the effect of indomethacin on T cell responses has not been studied in TB infection.

The objective of this study was to analyze COX-2 expression in monocytes from patients with latent and active TB and to explore the in vitro effects of the COX-i indomethacin on *Mtb*-specific T cell responses and regulation. We show that indomethacin down-regulates *Mtb* antigen stimulated Tregs, antigen induced cytokine responses, in particular TNF-α+ cells, and T cell proliferation. Our data suggest a potential role for COX-i in modulation of immune responses in TB infection. However, the functional consequences of our data need to be further evaluated and the potential for COX-i as HDT in TB infection should be explored in both animal studies and in human clinical trials.

## Methods

### Study participants

Ten patients with active TB disease (ATB) and nine patients with latent TB (LTB) were recruited from Haukeland University hospital, Bergen, Norway (cohort A). Additional, 23 patients with active TB disease were recruited from Oslo University hospital, Oslo, Norway (cohort B). Table 1 summarizes demographic and clinical patient characteristics. All patients were HIV uninfected. The diagnosis of active TB was based on a positive *Mtb* culture or on clinical and radiological findings. Subjects with a positive QuantiFERON®-TB test and with no signs of active TB based on X-ray, sputum or biopsy examination and clinical evaluation were defined as LTB.

**Table 1:** Patient characteristics

	Cohort A		Cohort B
	ATB (*n* = 10)	LTB (*n* = 9)	ATB (*n* = 23)
Age (median years, range)	28 (16–72)	43 (26–67)	29 (20–60)
Female (%)	3 (30)	4 (44)	9 (39)
Origin (%)			
Africa	4 (40)	4 (44)	10 (43)
Asia	2 (20)	2 (22)	10 (43)
Europe	4 (40)	3 (33)	3 (14)
Localisation (%)			
Pulmonary	10 (100)	-	17 (74)
Extrapulmonary^a^	-	-	6 (26)
ESR^b^ (median mm/hour, range)	42 (21–77)	16 (7–23)	21 (1–103)

Characteristics of patients included at Haukeland University hospital, Bergen, Norway (cohort A) and Oslo University hospital, Oslo, Norway (cohort B)

*ATB* active TB, *LTB* latent TB

^a^glandular, pericardial, pleural

^b^Erythrocyte Sedimentation Rate

### Sample processing

**Fig. 1:**
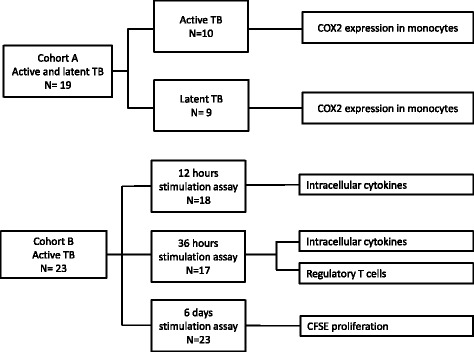
Flowchart of patient samples. Patients were included at two study locations. Cohort A (latent TB and active TB) denotes patients included at Haukeland University Hospital, whilst cohort B denotes patients included at Oslo University Hospital. The figure also shows the distribution of patient samples in the different assays performed in the study

Blood samples were obtained before initiation of anti-TB chemotherapy. Peripheral blood mononuclear cells (PBMCs) from participants recruited in cohort A were isolated using density gradient centrifugation (Lymphoprep™, Fresenius Kabi Norge AS, Halden, Norway), cryopreserved in 10 % DMSO/90 % fetal bovine serum (FBS, GIBCO, Life technologies) and stored in liquid nitrogen until analysis. PBMCs from participants recruited in cohort B were isolated in cell preparation tubes (CPT) (Becton Dickinson, BD, New Jersey, USA) with sodium heparin and cryopreserved in 90 % fetal calf serum (FCS, Sigma, Missouri, USA)/10 % DMSO and stored at −145 °C until analysis. Participants recruited in cohort A were only included in the monocyte assay and participants recruited in cohort B were only included in the Treg, proliferation and intracellular cytokine assays. Therefore, all assays only included PBMCs isolated by either of the methods. Figure 1 gives an overview of the number of samples used in the different assays in the study.

### Detection of COX-2 in monocytes

Cryovials from active and latent TB patients were thawed, washed and resuspended in RPMI-1640 media (Sigma-Aldrich) with 10 % FBS. PBMCs with viability <70 % were excluded from further analyses. After resting 6 h, PBMCs were allocated to a monocyte assay for 12 h stimulation with Lipopolysaccharide (LPS) (10 ng/mL, Sigma-Aldrich) or unstimulated RPMI media. The cells were stained with Live/Dead discriminator, followed by cell surface staining of CD3, CD4, CD14, CD16 and HLA-DR and intracellular staining of COX-2. The following live/dead discriminator stain and directly conjugated monoclonal antibodies were used in the monocyte assay: Live/Dead Fixable Near-IR Dead Cell Stain kit (Life technologies), anti-CD3-PE-Cy7, anti-CD4-Horizon V500, anti-CD14-APC, anti-CD16-PE, anti-HLA-DR-BV421 (all from BD Bioscience) and anti-COX-2 (Cayman Chemicals, Ann Arbor, MI, USA). Human FOXP3 buffer set (BD bioscience) was used for fixation and permeabilisation.

### T regulatory cells and intracellular cytokine assays

PBMC’s (0.75 -1 × 10^6^ cells/well) from patients with untreated active TB were rested 6 h before stimulation with *Mtb* derived 6 kDa early secretory antigenic target (ESAT-6, 2 ug/ml) and Antigen 85 (Ag85, 2 ug/ml) complex (both 15mer overlapping peptide, Genscript, HK limited; >85 % purity). The samples were stimulated with or without the COX-i indomethacin (25 μM, Sigma Aldrich). Staphylococcal enterotoxin B (SEB) (1 ug/ml, Sigma-Aldrich) was used as positive control and serum-free medium (AIM V; Gibco Invitrogen, Carlsbad, CA, USA) with 0.1 % highly purified human albumin as negative control. Due to limited number of cells indomethacin was not added to the SEB stimulated samples. Only PBMCs with viability >80 % were included. Samples with COX-i were pre-treated with indomethacin 2 h before stimulation and then incubated for 12 and 36 h. In the 12 h assay, Brefeldin A (BFA), final concentration 10 ug/ml (BD Bioscience) was added at time of stimulation, whilst in the 36 h assay BFA was added for the last 10 h to avoid prolonged incubation with potential toxic effects of BFA [32]. In both the 12 and 36 h assay, the cells were washed and stained with live/dead discriminator in azide-free and serum/protein-free PBS followed by CD4, CD3 and CD25 surface staining. The samples were washed and fixed/permeabilized with the FOXP3 staining kit (BD Biosciences) according to the manufacturer’s instructions. Subsequently, cells were stained for intracellular TNF-α, IFN-γ, IL-2 and FOXP3. The following reagents were used in the 12 and 36 h stimulation assays for detection of intracellular cytokines and Tregs: anti-CD4- APC- H7, anti-CD3-PerCP-Cy5.5, anti-CD25-BV605, anti-IL-2-PE, anti-IFN-γ- PE-Cy7, anti-TNF-α- APC, anti-FOXP3- AF 488 (all from BD Bioscience) and Fixable Viability Dye eFluor® 450 (eBioscience, San Diego, USA).

### CFSE proliferation assay

PBMCs (5 × 10^5^cells/well) from active TB patients were thawed and rested 6 h before labeling with Carboxyfluorescein succinimidyl ester (CFSE) according to manufacturer’s procedure. In the COX-i treated samples, indomethacin was added 2 h prior to stimulation with ESAT-6 and Ag85. The samples were incubated for 6 days and then washed and stained for surface markers and viability staining. Fluorochromes used in the 6 days proliferation assay; anti-CD3-V450, anti-CD4- APC H7, anti-CD45RA -BV 605, anti-HLA DR- APC, anti-CD25-PE, anti-CD127- PeCy7 and 7AAD- PerCP (all from BD Bioscience) and CellTrace™ CFSE Cell Proliferation (Life technologies).

### Flow cytometry analyses

Flow cytometric acquisition was performed on a BD FACS Canto II and a BD LSR Fortessa flow cytometer. At least 10.000 CD4+ and CD8+ T cells were analyzed for the intracellular analyses of cytokines and FOXP3 expression in T cells and minimum 1.000 monocytes were required for the analyses of COX-2 expression in monocytes. FlowJo version 10 (TreeStar Inc, Ashland, OR, USA) was used for data analyses. Dead cells were excluded from the lymphocyte and monocyte populations before applying the different gating strategies. In the cytokine analyses, frequencies (percentage of parent population) of *Mtb* antigen stimulated cytokine producing T cells were calculated. Total IFN-у+, IL-2+ or TNF-α+ describe all CD4+ or CD8+ cells positive for the cytokine measured, while Boolean gating strategy was used to create cytokine combinations defined as: polyfunctional (IFN-у+IL-2+TNF-α+), double positive (IFN-у+IL-2+ or IL-2+TNF-α+or IFN-у+TNF-α+) and single positive (IFN-у+ or IL-2+ or TNF-α+) producing CD4+ and CD8+ T cells. Tregs were defined as FOXP3+CD25++CD4+ or as FOXP3+ CD45RA-CD4+ T cells [33]. In the T cell proliferation assay, cut-off for proliferating cells was set to the peak of the 2nd generation of CFSE^dim^ CD4+ or CD8+ T cells. Frequencies (percentage of parent population) of *Mtb* antigen-stimulated cytokine-producing T cells, Tregs and proliferation T cells are all shown without subtracting background values (unstimulated controls) to better delineate the effect of indomethacin on stimulated versus unstimulated samples. Monocytes were defined as “true monocytes” after exclusion of CD3+ and CD16+HLA-DR- cells [34] and COX-2 expression (percentage of parent population) by true monocytes was analysed. The statistical region in the monocyte population was set by use of COX-2 human blocking peptide (Cayman chemicals) according to the manufacturer’s instructions.

### Statistical analyses

Statistical analyses were performed by SPSS statistics 22 (IBM) and Statistica v 7.0 (Statsoft, Tulsa, OK, USA). Non-parametrical statistical methods were applied. For group-wise comparison Mann-Whitney U test was applied and for dependent variables the two-tailed Wilcoxon matched pair test. A significance level of 0.05 was used. All values are presented as median and interquartile range [IQR]. Graphical presentations were made using Prism V5.04 and V6 software (GraphPad, San Diego, USA).

## Results

### COX 2 expression in monocytes in active and latent tuberculosis

**Fig. 2:**
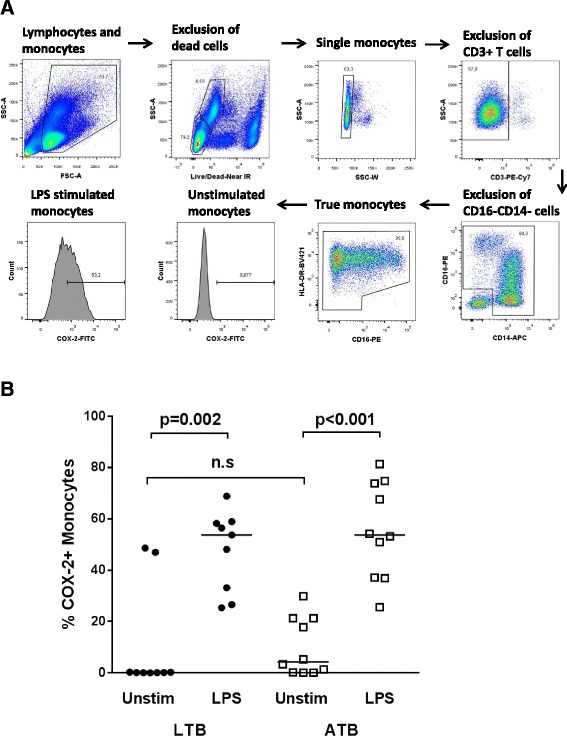
COX-2 expression in monocytes. **a** Gating strategy for identification of true monocytes [34]. **b** Comparison of COX-2 expression in unstimulated true monocytes in PMBCs from patients with latent TB (LTB, *circles*) and active TB (ATB, *squares*). PBMCs from patients with LTB and ATB were left unstimulated (Unstim) or stimulated for 12 h with LPS. P- values were calculated by Wilcoxon matched pairs test for paired samples and Mann- Whitney U test for group wise comparison (n.s = non-significant). *Horizontal lines* in **b** represent median values

We first analyzed COX-2 levels in monocytes from patients with latent or active TB (gating strategy, Fig. 2a). Although not significant, unstimulated monocytes from patients with active TB tended to express higher levels of COX-2 compared to patients with latent TB (Fig. 2b). Still, monocytes from both TB groups were able to significantly up-regulate COX-2 expression after 12 h in vitro LPS stimulation (Fig. 2b).

### Indomethacin reduces up-regulation of *Mtb* antigen induced FOXP3+CD25++ Tregs

**Fig. 3:**
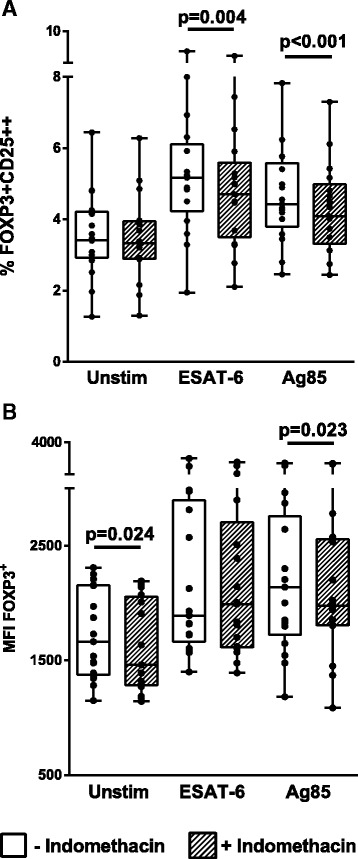
Effect of indomethacin on TB antigen induced FOXP3+CD25++ CD4+ Tregs. PBMCs were obtained from patients with active TB (*n* = 17) prior to treatment and left unstimulated (unstim) or stimulated for 36 h with ESAT-6 or Ag85 without (open boxes) or with (hatched boxes) addition of indomethacin. The figures show percentages of FOXP3+CD25++ (**a**) and FOXP3 median fluorescence intensity (MFI) (**b**) in the CD4+ cells. P- values were calculated by Wilcoxon matched pairs test. Plots are shown as *box plots* together with individual data points with median, IQR and minimum/maximum values. Significant changes between stimulated samples with and without indomethacin are denoted with *p*-values in figure. Levels of FOXP3+CD25++ CD4+ T cells and FOXP3 MFI were significantly upregulated upon stimulation with both E6 and Ag85 (*p* < 0.01, values not shown in figure)

We then assessed the in vitro effects of the COX-i indomethacin on Tregs from patients with active TB disease prior to initiation of anti-TB chemotherapy (Fig. 3). FOXP3+CD25++ Tregs were analyzed after 36-h TB antigen stimulation, shown to be the optimal time for analyzing FOXP3 changes in Tregs [35]. We observed a significant up-regulation both in the fraction and the median fluorescence intensity (MFI) of FOXP3+CD25++ Tregs in the ESAT-6 and Ag85 stimulated CD4+ T cells with a significant reduction in the samples treated with indomethacin both for the fraction of FOXP3+CD25++ Tregs (ESAT-6; *p* = 0.004 and Ag85; *p* < 0.001) (Fig. 3a) and FOXP3 MFI (unstim; *p* = 0.024 and Ag85; *p* = 0.023) (Fig. 3b).

### Indomethacin reduces *Mtb* antigen induced T cell cytokine production

**Fig. 4:**
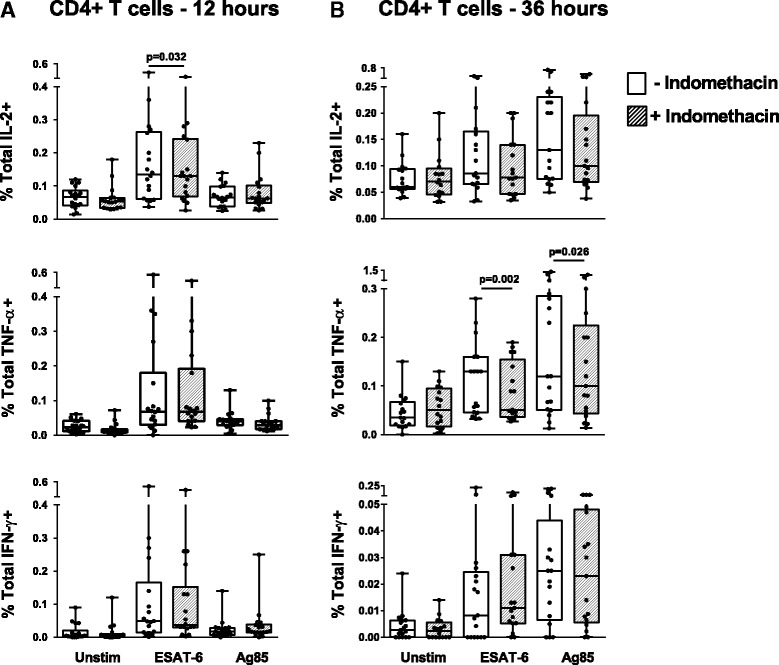
Effect of indomethacin on TB antigen induced intracellular cytokines in CD4+ T cells. Total IFN-у+, IL-2+ and TNF-α+ T cell responses in unstimulated (Unstim), ESAT-6 and Ag85 stimulated PBMCs without (*open boxes*) and with (*hatched boxes*) indomethacin after 12 h (**a**, *n* = 18) and 36 h (**b**, *n* = 17) stimulation. PBMCs were obtained from patients with active TB prior to treatment. *P*- values calculated by Wilcoxon matched pairs test. Plots are shown as *box plots* with individual data points with median, IQR and minimum/maximum values. Significant changes between stimulated samples with and without indomethacin are denoted with p-values in figure

To assess the effect of indomethacin on *Mtb* specific CD4+ T cells, we stimulated cells with *Mtb* peptides with or without addition of indomethacin and measured intracellular cytokine production after 12 and 36 h. There were significant up-regulation of CD4+ T cell subsets producing both IL-2, TNF-α and IFN-γ, in ESAT-6 stimulated cells already after 12 h and in Ag85 stimulated samples also after 36 h (Fig. 4). Indomethacin reduced the fraction of total IL-2 producing cells after 12 h (ESAT-6; *p* = 0.032) (Fig. 4a) and the fraction of total TNF-α producing cells after 36 h stimulation (ESAT-6; *p* = 0.002 and Ag85; *p* = 0.026) (Fig. 4b), whereas no significant changes were seen for the IFN-γ producing cells.

**Fig. 5:**
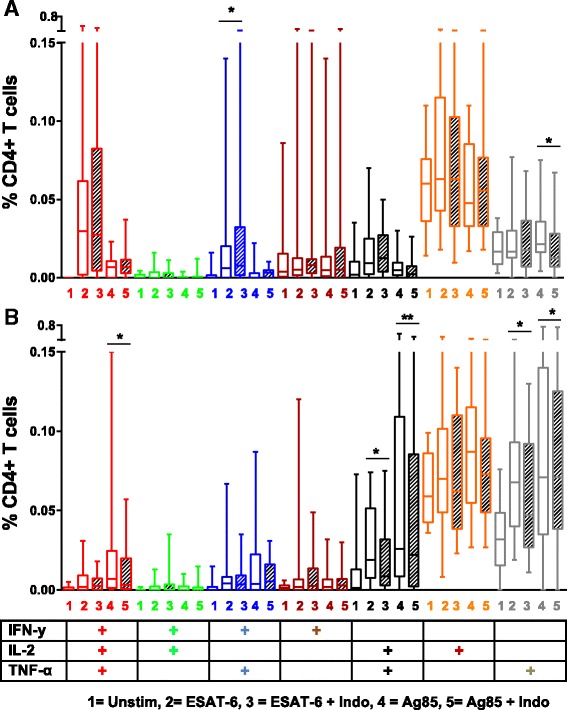
*Mtb* antigen induced single, duo and polyfunctional cytokine producing CD4+ T cells. PBMCs were obtained from patients with active TB prior to treatment and left unstimulated or stimulated with ESAT- 6 or Ag85 without (*open boxes*) or with indomethacin (*hatched boxes*) after 12 h (**a**) and 36 h (**b**) stimulation (*n* = 18 and *n* = 17 respectively). Boolean gating strategy was used to create cytokine combinations of single-producing, duo-producing and polyfunctional T cells. *P*- values were calculated by Wilcoxon matched pairs test ( *p <0.05, **p < 0.01). Plots are shown as *box plots* with median, IQR and minimum/maximum values

Single, double and polyfunctional CD4+ T cells were also determined. After 12 h of stimulation there was no clear pattern in response to indomethacin (Fig. 5a). The TNF-α+IFN-γ+CD4+ cells demonstrated a slight increase (ESAT-6; *p* = 0.023), whilst single TNF-α+ cells decreased (Ag85; *p* = 0.043). However, after 36 h stimulation, we observed a more distinct pattern in cells treated with indomethacin with down-regulation of the following cytokine producing CD4+ T cell subsets; IFN-γ+IL-2+TNF-α+T cells (Ag85; *p* = 0.030), IL-2+TNF-α+(ESAT-6; *p* = 0.046 and Ag85; *p* = 0.007) and single-TNF-α+ cells (ESAT-6; *p* = 0.011 and Ag85; *p* = 0.031) (Fig. 5b).

CD8+ T cells also significantly up-regulated their cytokine production upon *Mtb* antigen stimulation, but the effect of indomethacin was less pronounced. Overall there was a decrease in cytokine production, but only significant for total IFN-γ+ and polyfunctional IFN-γ-+IL-2+TNF-α+CD8+ T cells after 36 h stimulation (ESAT-6; *p* = 0.039 and *p* = 0.017, respectively) (data not shown).

### Indomethacin modulates *Mtb* antigen induced T cell proliferation

**Fig. 6:**
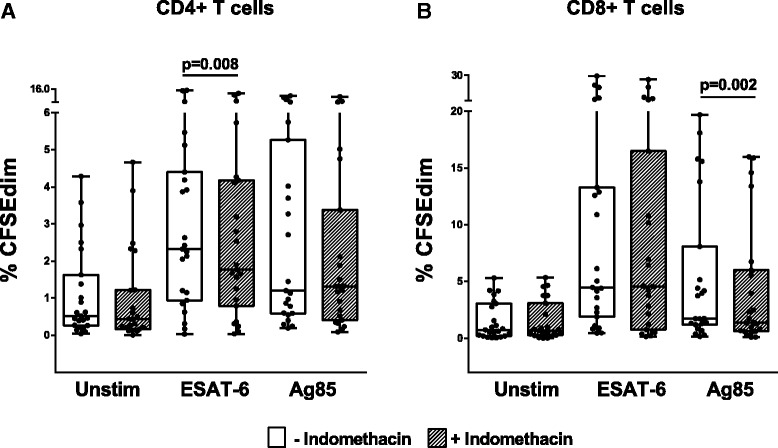
Effect of Indomethacin on CD4+ and CD8+ T cell proliferation. Proliferative responses measured by percentages of CFSE^dim^ in unstimulated, ESAT-6 and Ag85 stimulated PBMCs without (*open boxes*) and with (*hatched boxes*) indomethacin after 6 days stimulation. PBMCs were obtained from patients with active TB prior to treatment. **a** CD4+ T cells (*n* = 23). **b** CD8+ T cells (*n* = 23). *P*- values were calculated by Wilcoxon matched pairs test. Plots are shown as *box plots* with individual data points with median, IQR and minimum/maximum values. Significant changes between stimulated samples with and without indomethacin are denoted with *p*-values in figure. %CFSEdim cells were significantly upregulated upon stimulation with both E6 and Ag85 (*p* < 0.001, values not shown in figure)

The proliferative capacity of *Mtb* peptide stimulated CD4+ and CD8+ T cells was assessed by CFSE staining in a 6 days stimulation assay. There was a significant increase in both proliferating CD4+ (ESAT-6 and Ag85; *p* < 0.001) and CD8+ T cells (ESAT-6 and Ag85 *p* < 0.001) in stimulated compared to unstimulated cells (Fig. 6). A significant, but modest, decrease of proliferating CD4+ T cells was observed in the indomethacin treated ESAT-6 stimulated samples (*p* = 0.008) (Fig. 6a), whilst no significant changes were seen in the Ag85 stimulated samples. For the CD8+ subsets, indomethacin likewise reduced proliferation of Ag85 stimulated cells (*p* = 0.002) (Fig. 6b). Cell surface activation markers were also analyzed after 6 days stimulation and showed a decrease in the fraction of activated CD25+HLA-DR+ (ESAT-6; *p* = 0.011 and Ag85; *p* = 0.044) CD4+T cell subsets in response to indomethacin treatment (data not shown).

## Discussion

Host-directed immune modulating therapies have been suggested as adjunct treatment in combination with standard anti-TB antibiotics. Anti- inflammatory drugs such as corticosteroids are already in use as adjunctive therapy in TB meningitis with reduction of both morbidity and mortality [36]. Non-steroid Anti- inflammatory drugs (NSAIDS) are already in use to relieve symptoms in non-severe cases of paradoxical TB-IRIS [37] and there are anecdotal reports of use of NSAIDS in complicated cases of TB disease, however such practice is poorly documented in literature. In this study we explored the in vitro effects of the COX-i indomethacin on immune cells obtained from patients with active TB disease prior to initiation of anti-TB chemotherapy. We demonstrate for the first time that indomethacin significantly down-regulates the fraction of *Mtb* antigen induced Tregs as well as T cell proliferation and TNF-α cytokine production.

First, we explored COX-2 expression in monocytes, the major source for PGE2 production in a small cohort of active and latent TB infection. Monocytes from patients with active TB exhibit functional and phenotypical alterations compared with healthy controls [38, 39] that could be restored by TB treatment [40]. Different species of mycobacteria have been suggested to induce expression of COX-2 through different signaling pathways [41]. To our knowledge, there are no previous studies comparing COX-2 expression in latent versus active TB in humans. Interestingly, we found a tendency of higher spontaneous expression of COX-2 in the unstimulated active TB compared to the latent TB samples, possibly indicating an ongoing activation of the COX-2-PGE2 pathway in patients with active disease. However, as shown by our data, also in otherwise healthy patients with latent TB high levels of COX-2 could be expressed. Thus, COX-2 levels in monocytes at different stages of TB infection and any implication for disease progression needs to be further studied in larger cohorts.

The main finding in our study was the reduction of antigen induced Tregs in the indomethacin treated samples. Of note, indomethacin also reduced the levels of FOXP3 MFI in the unstimulated samples, although to a lesser extent than seen in the stimulated samples. This possibly reflects an up-regulated COX expression and increased PGE2 production in vivo in patients with active TB. There are several reports of increased levels of Tregs in TB disease [42–44], but the role of these immune regulatory cells during chronic TB infection is unclear [45]. Still, experience from animal models has proven detrimental effects of Tregs during the initial stage of infection [46]. Treg mediated suppression of effector T cells occurs through different mechanisms [47] and Tregs may inhibit T cell effector functions in a COX-2-PGE2 dependent manner [14]. We find support for the use of COX-i in targeting Tregs from studies showing that PGE2 induces FOXP3 gene expression and Treg function in human CD4+ T cells [48]. Both the up-regulation of FOXP3 in Tregs and their suppressive effect on effector T cells have been shown to be reversed by COX- i [14]. To our knowledge there are no published data of immune therapy aiming to modulate Tregs in human TB disease. There is however an ongoing phase I/II clinical trial on COX-2-i in active TB where Tregs will be studied (ClinicalTrials.gov Identifier: NCT02503839). An additional clinical trial aims to reduce the risk of developing active TB by oral supplementation of a probiotic containing heat-killed environmental mycobacteria (ClinicalTrials.gov Identifier: NCT02076139). Pre-clinical animal models to this study have shown that the mycobacteria delay progression to active TB in mice by inducing PPD specific Treg populations [49]. Nevertheless, we claim that our observed reduction of FOXP3 in indomethacin treated Tregs may be beneficial to the host, shifting the balance towards a more effective immune response in long-lasting and advanced TB disease.

Animal models also support PGE2 as a significant factor in the pathogenesis of a dysfunctional hyperactivated immune system in TB, concluding with beneficial effects of COX-i with suppressed PGE2 concentrations; reductions in pulmonary inflammation and bacillary load, reversal to a Th1 cell profile and improved survival of TB infected mice [21–24]. In contrast, one study reports reductions in morbidity and mortality by augmenting PGE2 levels in *Mtb*-infected mice [25]. The conflicting reports may be due to differences in time since TB infection, emphasizing the need to be aware of differential effects of PGE2 in recently versus chronically TB infected mice.

Both CD4+ and CD8+ cytokine producing T cells play a major role in protection and immunity against TB disease [50], but contrasting results have made it difficult to define protective TB immunity based on cytokine responses [51–53]. In previous clinical trials, we have shown that HIV infected patients on antiretroviral therapy experience an improved immune response with concomitant treatment with COX2-i [26–28]. In the present study, the COX-i indomethacin modestly reduced the *Mtb* antigen induced CD4+ and CD8+ cytokine responses as well as the proliferative capacity of the T cells. Our data show that in particular the CD4+TNF-α+ cell subsets were significantly reduced when treated with indomethacin, but still with detectable levels. Experience from animal models show that TNF-α is important for control of TB [54, 55] and treatment with TNF-α blockers have been associated with the progression from latent to active TB in humans [56, 57]. TNF-α is essential for maintenance and formation of the granuloma, but excess TNF-α production also contributes to increased inflammation and pathology in TB [58]. Thus, one of the purposes of immune modulating therapy may be to decrease pathology related to excess TNF-α production while maintaining adequate TNF-α levels necessary for containment of *Mtb* [59]. Accordingly, the decreased levels of TNF-α producing CD4+ T cells observed in our study may constitute a potential beneficial response in a setting with chronic untreated TB disease.

The observed reduction of cytokines and proliferative capacity of T cells may be due to the direct effects of indomethacin inhibiting activation of the intracellular NF-κB pathway [60]. The NF-κB pathway is a key mediator of genes involved in the control of cellular proliferation and apoptosis [61]. Inhibition of the NF-κB pathway may be involved in the anti-inflammatory as well as the growth inhibitory properties of certain COX-i [62, 63] and COX-i have also been reported to inhibit NF-κB activation in cell culture [64]. In TB, reports have shown that TNF-α-induced NF-κB signaling pathway is central to the *Mtb-*specific immune response, and regulation of intracellular NF-κB signaling dynamics may be a key to control TB infection [65]. Thus, in summation, one must consider that COX-i may exert a relatively stronger inhibitory effect on the Th1 effector cells than the anticipated beneficial indirect effect following reduced Treg numbers.

There are limitations to our study. First, the relatively small number of patients gives reduced power in the statistical calculations and increases the risk for Type II statistical errors. Second, multiple testing increases the risk of type I errors. As there is no gold standard for how to handle multiple comparisons when considering partially dependent variables a significance level of 0.05 was used. Due to limited numbers of samples and PBMC’s available, we were not able to perform sorting of Tregs with more in-depth mechanistic studies of the potential impact of the observed decrease of indomethacin treated Tregs. For the same reason all analysis could not be performed in the latent TB group. Thus, future studies on COX-i should include latent TB controls and also investigate the *in vivo* effects of COX-i in clinical trials of patients in different stages of *Mtb* infection.

## Conclusions

In conclusion, our data indicate that indomethacin may be used to modulate immune responses in TB infection by reducing the fraction of *Mtb* specific Tregs with a concomitant reduction of *Mtb* specific cytokine responses and T cell proliferation in active TB disease. Still, the effects of COX-i in TB infection need further evaluation in human models and future clinical trials should be performed to explore the effects of COX-i as HDT options in different settings of TB infection and disease.

## Abbreviations

COX: Cyclooxygenase
COX-i: Cyclooxygenase inhibitor/s
HDT: Host-directed therapies
LPS: Lipopolysaccharide
MDR-TB: Multi drug resistant TB
MFI: Median fluorescence intensity
*Mtb*: Mycobacterium tuberculosis
PBMC(s): Peripheral blood mononuclear cells
PGE2: Prostaglandin E2
SEB: Staphylococcal enterotoxin B
TB: Tuberculosis
